# Impact of Experimental Hookworm Infection on the Human Gut Microbiota

**DOI:** 10.1093/infdis/jiu256

**Published:** 2014-05-03

**Authors:** Cinzia Cantacessi, Paul Giacomin, John Croese, Martha Zakrzewski, Javier Sotillo, Leisa McCann, Matthew J. Nolan, Makedonka Mitreva, Lutz Krause, Alex Loukas

**Affiliations:** 1 Department of Veterinary Medicine, University of Cambridge, United Kingdom; 2 Australian Institute of Tropical Health and Medicine, Queensland Tropical Health Alliance Laboratory, James Cook University, Cairns; 3 Prince Charles Hospital; 4 Bioinformatics Laboratory, QIMR Berghofer Medical Research Institute, Brisbane, Australia; 5 Royal Veterinary College, University of London, Hawkshead, United Kingdom; 6 The Genome Institute; 7 Department of Medicine, Washington University School of Medicine, St. Louis

**Keywords:** experimental infection, hookworms, parasitic helminths, 16S rRNA gene, pyrosequencing, chronic inflammatory disorders, microbial richness

## Abstract

The interactions between gastrointestinal parasitic helminths and commensal bacteria are likely to play a pivotal role in the establishment of host-parasite cross-talk, ultimately shaping the development of the intestinal immune system. However, little information is available on the impact of infections by gastrointestinal helminths on the bacterial communities inhabiting the human gut. We used 16S rRNA gene amplification and pyrosequencing to characterize, for the first time to our knowledge, the differences in composition and relative abundance of fecal microbial communities in human subjects prior to and following experimental infection with the blood-feeding intestinal hookworm, *Necator americanus*. Our data show that, although hookworm infection leads to a minor increase in microbial species richness, no detectable effect is observed on community structure, diversity or relative abundance of individual bacterial species.

The complex interactions that occur between pathogenic microorganisms and commensal bacteria are crucial for the establishment of host-pathogen cross-talk, ultimately shaping the development of the intestinal immune system [1]. In the developed world, perturbation of the fine balance between the human host, parasites, and gut bacteria, following a steady decline in prevalence of gastrointestinal helminth infections, has been associated with an increased susceptibility to allergic and autoimmune diseases of the gastrointestinal tract, such as Crohn's disease and Coeliac disease (CeD)–this is embodied by the “hygiene hypothesis” [2]. Building on this observation, several attempts have been made to re-establish this balance by reintroducing gastrointestinal helminths into patients as a novel immunotherapy [3–5]. A range of experimental and observational studies support a key role for gastrointestinal nematodes in promoting the development of regulatory responses in the host gut, which ultimately results in an amelioration of the pathology of chronic inflammation [3, 6]. For instance, experimental infection of human volunteers with the blood-feeding hookworm, *Necator americanus*, results in up-regulation of anti-inflammatory cytokines, such as interleukin 10 (IL-10) which, in turn, is hypothesized to play a pivotal role in the suppression of immune hyper-responsiveness [7]. The ability of gastrointestinal parasitic helminths to modulate the immune system of the host is therefore likely to represent the key mechanism by which parasites are able to suppress the development of inappropriate responses; however, the involvement of other clues, such as individual and environmental factors, cannot be excluded.

Several studies have highlighted the pivotal roles that disturbances in the commensal intestinal microbiota play in the dysregulation of the immune system, thus leading to the development of chronic inflammatory disorders of the intestinal tract [1]. It is therefore conceivable that one of the mechanisms by which parasitic helminths modulate intestinal inflammation is *via* alteration of the composition of the gut microbiota. Consistent with this hypothesis, a recent study [8] using a primate model of idiopathic chronic diarrhea has demonstrated that the therapeutic ability of *Trichuris* whipworms to improve clinical symptoms of chronic inflammation was associated with significant changes in the relative abundance of several commensal bacterial species within the gut. However, it is currently unknown whether similar mechanisms occur during helminth infections in humans. Indeed, although recent studies have investigated the potential therapeutic properties of *N. americanus* for Crohn's disease and CeD [4, 5], the effects that experimental, low-dose *N. americanus* infections exert on the intestinal microbiota are yet to be assessed. Hence, in the present study, we examined the effects of experimental hookworm infection of human volunteers on the composition of the fecal microbiota using 16S rRNA pyrosequencing and bioinformatic analyses of sequence data.

## METHODS

This study was approved and carried out in strict accordance and compliance with the National Statement on Ethical Conduct in Research Involving Humans guidelines of the National Health and Medical Research Council of Australia (www.nhrmc.gov.au/publication/humans/contents.htm; www.nhmrc.gov.au/funding/policy/researchprac.htm). The Prince Charles Hospital (Brisbane, Australia) and James Cook University Human Research Ethics Committees approved the study. Written informed consent was obtained from all subjects enrolled in the study. None of the subjects had received any antibiotic treatment prior to sample collection. This study was registered as a clinical trial at ClinicalTrials.gov (NCT00671138). Briefly, 8 otherwise healthy volunteers with HLA-DQ2+ CeD on a long-term gluten-free diet (since >6 months) were recruited and infected percutaneously with 20 infective third stage larvae (iL3) of *N. americanus* [5]. Prior to experimental infection (T0), as well as at 8 weeks post-infection (T8), individual fecal samples (approximately 10 g each) were collected from each subject and stored at −20°C until subsequent DNA extraction. Genomic DNA was extracted directly from each sample using the PowerSoil DNA isolation kit (MoBio, USA), according to the manufacturer's instructions. Parallel sequence data sets were generated for individual DNA samples by sequencing of 2 separate fragments of the 16S rRNA gene that encode the V1-V3 and the V3-V5 hypervariable regions on a 454 GS-FLX Titanium System (Roche) using universal primers described elsewhere [9, 10]. Forward primers incorporated GS Titanium adapters as well as a sample-specific barcode sequence. Raw sequence data have been deposited in the NCBI Sequence Read Archive under study accession number SRP041283.

Sequence data were processed using the Quantitative Insights Into Microbial Ecology (QIIME) software suite [11]. Briefly, after filtering of low-quality reads, all remaining sequences were de-multiplexed according to barcode, including error-correction to reduce the possibility of sample misassignment. Chimeric sequences were removed using UCHIME v 3.0.617. Sequences were subsequently clustered into operational taxonomic units (OTUs) on the basis of similarity to known bacterial sequences in the Greengenes database (http://greengenes.secondgenome.com/; cut-off: 97% sequence similarity) using the UCLUST software; then, sequences were assigned to taxonomy using the Ribosomal Database Project (RDP) Classifier with the confidence level set at 0.8. Statistical analyses and data mining were conducted using the Calypso software (http://bioinfo.qimr.edu.au/calypso/). All analyses were conducted separately for each data set (V1–V3 and V3–V5). Shannon diversity and richness were compared by paired *t*-test and rarefaction curves. Differences in abundance of individual OTUs were assessed by paired *t*-test on relative abundance values, and *P* values were corrected for multiple testing by false discovery rate (FDR). Anosim (Jaccard distance), redundancy analysis (RDA; including human subject and week as explanatory variables), principal coordinates analysis (PCoA; Jaccard distance), and rarefaction analyses were run in Calypso with default parameters. Anosim was run twice for each variable region, with samples labeled by infection status in the first run and by subject ID in the second run, respectively.

## RESULTS

**Figure 1.**
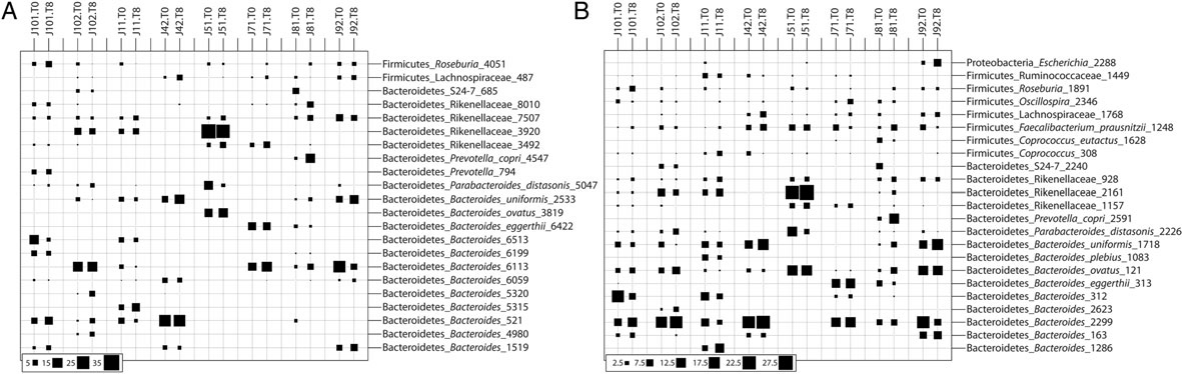
Composition of the fecal microbial communities in patients prior to and following infection by *Necator americanus* as predicted in the analysis of the V1–V3 (*A*) and V3–V5 (*B*) 16S rRNA gene. Bubble sizes reveal the relative abundance (%) of phylotypes (based on 97% sequence identity) in each sample. Codes represent individual subjects included in the study prior to (T0) and at week 8 (T8) post-infection.

**Figure 2.**
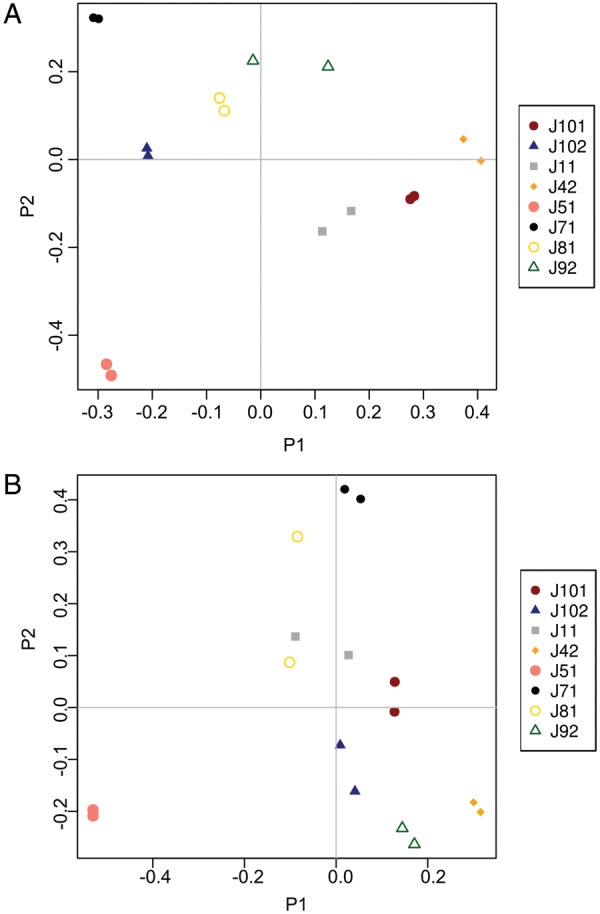
Principal coordinates analysis (PCoA) of the fecal microbiota of human subjects prior to and following experimental infection with *Necator americanus*, based on analyses of both V1–V3 (*A*) and V3–V5 (*B*) hypervariable regions of the prokaryotic 16S rRNA gene. Community similarity was calculated using the Jaccard distance measure of the phylotypes. Codes represent individual subjects included in the study prior to (T0) and at week 8 (T8) post-infection.

All subjects were positive for hookworm eggs in the feces (not shown), confirming successful intestinal infection with adult worms. A total of 81 825 (V1–V3) and 38 739 useable reads (V3–V5) were assigned to 9 and 7 bacterial phyla, respectively (Supplementary Table 1). Consistent with previous investigations, the phyla Bacteroidetes, Firmicutes, and Proteobacteria dominated the intestinal microbiota of all subjects included in the study both at T0 and T8 (Figure 1). A PCoA of the fecal microbial communities showed strong clustering of the samples by individual rather than infection status in the 2 main axes (Figure 2), thus indicating that the community composition of each subject remained stable over time. Similarly, both Anosim and RDA indicated a clear clustering of samples by individual (*P* < .002 Anosim; *P* < 1e^−5^ RDA for both V1–V3 and V3–V4). Hookworm infection did not impact community structure (*P* > .98 Anosim, *P* > .58 RDA for both V1–V3 and V3–V5). In line with this observation, there were no significant differences in relative abundance of any individual OTU identified in the study (97% sequence identity) between T0 and T8 (*P* > .67 for all detected OTUs, paired *t*-test, FDR adjusted). Albeit nonsignificant, at T8, an increase in bacterial species richness was observed (*P* = .07 for V3-V5; *P* = .1 for V1-V3) (Supplementary Figure 1); however, this did not impact Shannon diversity (*P* > .77 for both V1–V3 and V3–V5) or global community composition (*P* > .98 Anosim, *P* > .58 RDA for both V1–V3 and V3–V5).

## DISCUSSION

This study investigated, for the first time to our knowledge, the effect of experimental infection by a gastrointestinal human parasite on the bacterial species inhabiting the gut. The results indicate that acute hookworm infection does not have a major impact on the community structure of the intestinal microbiota. A limitation of our study is the relatively small sample size, which may have affected the statistical power and thus the ability to identify minor changes in the fecal microbiota following hookworm infection. However, our findings are supported by the results of a recent investigation conducted on a relatively large number of individuals [12], which reported no significant differences between the composition of the fecal microbiota of human subjects with naturally acquired whipworm (*T. trichiura*) infections and that of uninfected controls from the same region of Ecuador. In contrast, the results from our study and that by Cooper and colleagues [12] differ from the outcomes of similar preliminary investigations conducted in animals, in which gastrointestinal helminth infections (ie, *Heligmosomoides polygyrus* in mice and *T. suis* in pigs) were associated with significant changes in the composition of the gut microbiota [13, 14]. The differences observed between these studies may be attributed to the different helminth species under investigation and/or, in part, to the source of microbial material. Indeed, the experiments conducted in animals examined the microbiota within the intestinal tissue, which is generally unfeasible when working with human subjects. In particular, since the human volunteers enrolled in the present study were clinically healthy, the collection of biopsy specimens from these subjects was unjustifiable. Therefore, it is possible that hookworms and other helminths may induce modifications of the microbiota at the site of infection (eg, duodenal mucosa for hookworms) that are not reflected by the composition of the fecal microbial communities [12]. Another explanation for the lack of effect of *N. americanus* infection on the human fecal microbiota might be due to the intensity of infection. Indeed, in the present study, only 20 iL3s of *N. americanus* were administered percutaneously to each of the volunteers enrolled. Although this number may prove insufficient to induce detectable effects on the gut microbiota, it is considered sufficient to a regulatory immune response while remaining safe (J. Croese et al, unpublished data) [6, 7].

In the present study, hookworm infection was associated with a minor, albeit statistically insignificant, increase in bacterial richness (Supplementary Figure 1) Interestingly, studies investigating the association between the gut microbiota and inflammatory bowel disease have demonstrated that the species richness of the luminal commensal bacteria of symptomatic subjects is significantly lower than that of healthy controls [15]. We therefore suggest that, when administered to individuals with allergic or autoimmune disorders of the intestinal tract, hookworms exert their therapeutic potential, at least in part, by maintaining microbial species richness and thereby restoring microbial (and immune) homeostasis in the gastrointestinal tract. Consistent with this hypothesis, Broadhurst and colleagues [8] noted that differences between the gut microbiota of primates with symptomatic idiopathic chronic diarrhea prior to and following experimental whipworm infection might be attributed to the capacity of *Trichuris* to assist in the restoration of a normal, healthy gut flora. Whether controlled experimental hookworm infections may contribute toward restoring a healthy microbial flora in the intestinal tract of human patients affected by chronic inflammatory diseases remains to be addressed. Future studies could, for instance, focus on determining the differences between the composition of the gut microbiota, as well as the fluctuations in species richness, of a larger number of CeD subjects inoculated with hookworms prior to and following gluten challenge. This improved knowledge should assist the discovery of the full complement of biological interactions occurring at the hookworm-host interface and, in turn, the development of helminth-based therapeutics for allergic and autoimmune disorders.

## Supplementary Data

Supplementary materials are available at *The Journal of Infectious Diseases* online (http://jid.oxfordjournals.org). Supplementary materials consist of data provided by the author that are published to benefit the reader. The posted materials are not copyedited. The contents of all supplementary data are the sole responsibility of the authors. Questions or messages regarding errors should be addressed to the author.

Supplementary Data
