# Supplementary material for: Impact of Experimental Hookworm Infection on the Human Gut Microbiota
Source: J Infect Dis. 2014 May 3;210(9):1431–4. doi: 10.1093/infdis/jiu256 (PMC4195438; doi:10.1093/infdis/jiu256)
Supplement: Supplementary Data [file supp_210_9_1431__index.html]

Impact of Experimental Hookworm Infection on the Human Gut Microbiota — Impact of Experimental Hookworm Infection on the Human Gut Microbiota — Supplementary Data 

# Impact of Experimental Hookworm Infection on the Human Gut Microbiota

## Supplementary Data

Supplementary Data

**Files in this Data Supplement:**

- Supplementary Data - Doc file
- Supplementary Table 1 - xls file
