# Supplementary material for: Impact of Experimental Hookworm Infection on the Human Gut Microbiota
Source: J Infect Dis. 2014 May 3;210(9):1431–4. doi: 10.1093/infdis/jiu256 (PMC4195438; doi:10.1093/infdis/jiu256)

**Supplementary Figure 1**. Rarefaction curves depicting the overall taxonomic richness of the fecal microbiota of human subjects prior to and following experimental infection with *Necator americanus*, based on analyses of both V1-V3 (A) and V3-V5 (B) hypervariable regions of the prokaryotic 16S rRNA gene (clustering cut-off: 97% similarity). Each subject is color-coded, time-points are represented by symbols (circles: T0, triangles: T8).


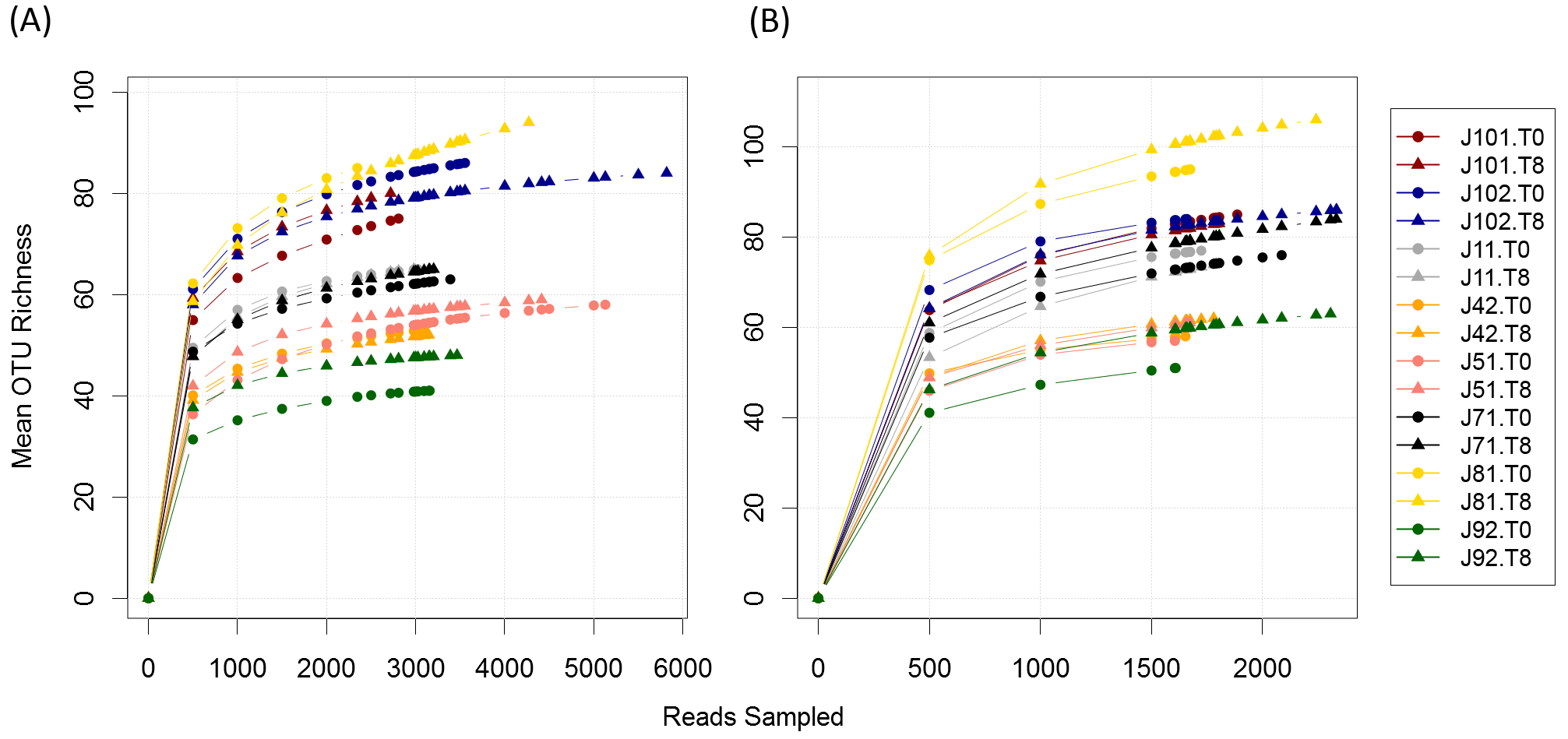

Supplement: Supplementary Data [file supp_jiu256_jiu256supp.doc]
